# Mitochondrial A‐kinase anchoring proteins in cardiac ventricular myocytes

**DOI:** 10.14814/phy2.15015

**Published:** 2021-09-13

**Authors:** Rinzhin T. Sherpa, Chase Fiore, Karni S. Moshal, Adam Wadsworth, Michael W. Rudokas, Shailesh R. Agarwal, Robert D. Harvey

**Affiliations:** ^1^ Department of Pharmacology University of Nevada Reno Nevada USA

**Keywords:** A kinase anchoring proteins, cAMP, mitochondria, ventricular myocytes

## Abstract

Compartmentation of cAMP signaling is a critical factor for maintaining the integrity of receptor‐specific responses in cardiac myocytes. This phenomenon relies on various factors limiting cAMP diffusion. Our previous work in adult rat ventricular myocytes (ARVMs) indicates that PKA regulatory subunits anchored to the outer membrane of mitochondria play a key role in buffering the movement of cytosolic cAMP. PKA can be targeted to discrete subcellular locations through the interaction of both type I and type II regulatory subunits with A‐kinase anchoring proteins (AKAPs). The purpose of this study is to identify which AKAPs and PKA regulatory subunit isoforms are associated with mitochondria in ARVMs. Quantitative PCR data demonstrate that mRNA for dual specific AKAP1 and 2 (D‐AKAP1 & D‐AKAP2), acyl‐CoA‐binding domain‐containing 3 (ACBD3), optic atrophy 1 (OPA1) are most abundant, while Rab32, WAVE‐1, and sphingosine kinase type 1 interacting protein (SPHKAP) were barely detectable. Biochemical and immunocytochemical analysis suggests that D‐AKAP1, D‐AKAP2, and ACBD3 are the predominant mitochondrial AKAPs exposed to the cytosolic compartment in these cells. Furthermore, we show that both type I and type II regulatory subunits of PKA are associated with mitochondria. Taken together, these data suggest that D‐AKAP1, D‐AKAP2, and ACBD3 may be responsible for tethering both type I and type II PKA regulatory subunits to the outer mitochondrial membrane in ARVMs. In addition to regulating PKA‐dependent mitochondrial function, these AKAPs may play an important role by buffering the movement of cAMP necessary for compartmentation.

## Graphical abstract

Protein kinase A (PKA) anchored to the outer membrane of mitochondrial in cardiac myocytes has previously been shown to play a critical role in slowing the diffusion of cAMP as it moves throughout the cytoplasm. The present study demonstrates that this effect may be due to type I as well as type II PKA associated with mitochondria through their interactions with the A‐kinase anchoring proteins D‐AKAP1, D‐AKAP2, and ACBD3.

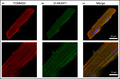

## 1 INTRODUCTION

Many different G protein‐coupled receptors are linked to the production of cyclic adenosine monophosphate (cAMP), a diffusible second messenger involved in regulating a multitude of responses in a variety of cell types. In cardiac myocytes, β‐adrenergic receptor (β‐AR) activation increases cAMP production, leading to the activation of effectors such as PKA. This is the canonical signaling pathway involved in controlling many aspects of excitation–contraction coupling in the heart (Bernstein et al., 2011). However, this simplistic description does not satisfy the question of how different receptors can elicit unique responses when they share this common second messenger. The classic example is in cardiac myocytes, where β‐ARs and E‐type prostaglandin receptors both stimulate cAMP production, but only β‐ARs elicit acute functional responses (Warrier et al., 2007). If cAMP produced by every GPCR were able to diffuse freely throughout the cell, it would uniformly activate PKA, independent of receptor location. But this is not always the case. To explain this behavior, it was originally hypothesized that cAMP signaling is compartmentalized (Brunton et al., 1979; Corbin et al., 1977; Hayes et al., 1979). More recently, direct demonstration of this phenomenon has been possible with the use of tools, such as fluorescence resonance energy transfer (FRET)‐based biosensors, that can detect cAMP activity in different subcellular locations (Agarwal et al., 2011, 2018; Nikolaev et al., 2004, 2006; Rudokas et al., 2021; Warrier et al., 2007; Zaccolo et al., 2000; Zaccolo & Pozzan, 2002). Studies using these methods have significantly advanced our understanding of the basis for this complex phenomenon. For example, the segregation of receptors into distinct membrane domains or subcellular locations contributes to differences in the compartmentation of cAMP produced by β_1_ and β_2_‐adrenergic receptors (Agarwal et al., 2011; MacDougall et al., 2012; Nikolaev et al., 2006; Rudokas et al., 2021) as well as β_1_ARs and E‐type prostaglandin receptors (Agarwal et al., 2011; Buxton & Brunton, 1983; Rochais et al., 2006; Warrier et al., 2007). However, this alone cannot explain compartmentalized cAMP signaling.

The movement of cAMP throughout the cell must also be restricted to generate compartmentalized responses. It has been suggested that the strategic placement of phosphodiesterases (PDEs), enzymes that break down cAMP, can act as functional barriers to its movement throughout the cell. While there is abundant evidence that PDEs are essential for creating localized differences in cAMP concentration (Jurevičius & Fischmeister, 1996; Leroy et al., 2008; Mongillo et al., 2004; Nikolaev et al., 2006; Zaccolo & Pozzan, 2002), they alone are not sufficient to prevent its free diffusion (Mongillo et al., 2004). Computational models have predicted that cAMP movement must be slowed by other factors before PDE activity can affect its spread, and recent experimental studies support that idea (Agarwal et al., 2016; Feinstein et al., 2012; Iancu et al., 2007; Lohse et al., 2017; Saucerman et al., 2006, 2014; Yang et al., 2016; Zhang et al., 2020). Consistent with this suggestion, we previously demonstrated that the movement of intracellular cAMP occurs at rates significantly slower than free diffusion, independent of PDE activity (Agarwal et al., 2016). Furthermore, we found that this behavior was due in large part to the binding of cAMP to PKA regulatory subunits specifically anchored to the outer membrane of mitochondria.

The PKA holoenzyme is a heterotetrameric structure consisting of two regulatory (R) and two catalytic (C) subunits. The binding of cAMP by the R subunits leads to the activation of the C subunits, which phosphorylate downstream substrates. The regulatory subunits also limit the ability of cAMP to move freely throughout the cell, especially when they are immobilized by A‐kinase anchoring proteins (AKAPs). AKAPs are scaffolding proteins that contain an amphipathic α‐helix that binds the regulatory subunits. They also possess sequences targeting them to specific subcellular locations. There are over 50 known AKAPs, at least 7 of which have been associated with mitochondria in one cell type or another (Diviani et al., 2016; Wong & Scott, 2004). The goal of the present study was to identify which AKAP and PKA regulatory subunit isoforms are associated with mitochondria in adult rat ventricular myocytes (ARVMs). In this report, we show that A‐kinase anchoring proteins D‐AKAP1, D‐AKAP2, ACBD3, and OPA1 are primarily mitochondrial. Moreover, all PKA regulatory subunit isoforms are associated with mitochondria, highlighting the importance of considering all isoforms when studying PKA‐dependent cAMP buffering in ARVMs.

## 2 MATERIALS AND METHODS

### 2.1 Adult cardiac myocyte isolation

ARVMs were isolated from the hearts of male Sprague Dawley rats (250–300 g) using enzymatic digestion and mechanical dispersion following a previously described procedure (Agarwal et al., 2011). The protocol used was in accordance with the *Guide for the Care and Use of Laboratory Animals* as adopted by the National Institutes of Health and approved by the Institutional Animal Care and Use Committee at the University of Nevada, Reno. Cells were used for experiments on the day of isolation.

### 2.2 RNA preparation and quantitative real‐time polymerase chain reaction (qPCR)

Total RNA was extracted from acutely isolated cardiac myocytes (Promega) following the manufacturer's instructions. Treatment with DNase was used to eliminate any gDNA contamination. RNA samples were then reverse transcribed using SuperScript IV Reverse Transcriptase (Life Technologies) to obtain cDNA. Real‐time PCR was carried out on the 7900HT Fast Real‐Time PCR system (Applied Biosystems) using 500 nM of primers (Table S1) with iTaq Universal SYBR Green supermix (Bio‐Rad) following the manufacturer's instructions. Primer specificity was verified by melt curve analysis. To determine the relative level of expression, each gene was normalized to the housekeeping gene, GAPDH, using the ΔΔC_T_ calculation (Danial et al., 2003; Livak & Schmittgen, 2001). Primer efficiencies for the set of primers ranged from 90% to 100%. Data are expressed as mean ± SEM.

### 2.3 Mitochondrial protein isolation and Western immunoblot analysis

The expression level of various AKAPs was measured in total cell lysates as well as cytosolic and mitochondrial fractions. Total cell lysates were prepared from acutely isolated cardiac myocytes. Briefly, cells were washed with ice‐cold PBS followed by incubation in RIPA lysis buffer on ice for 30 min. Cell lysates were then centrifuged at 4°C at 16,100 g for 15 min and the supernatants were collected for analysis. Cytosolic and mitochondrial fractions were obtained as described previously (Hom et al., 2010). Briefly, isolated myocytes were washed with cold isolation buffer (320 mM sucrose, 1 mM EDTA, and 10 mM Tris–HCl, pH 7.4) and collected by centrifugation at 700 g for 5 min. Cells were re‐suspended in isolation buffer containing protease inhibitor cocktail (Sigma‐Aldrich), homogenized, and centrifuged at 700 g for 10 min. The supernatant was then collected, and mitochondrial and cytosolic fractions were separated by centrifugation at 17,000 g. The pellet (mitochondrial protein fraction) was resuspended in lysis buffer (Cell Signaling Technology) containing 20 mM Tris–HCl (pH 7.5), 150 mM NaCl, 1 mM EDTA, 1 mM EGTA, 1% Triton X‐100, 0.2% SDS, 2.5 mM sodium pyrophosphate, 1 µM β‐glycerophosphate, 1 mM NaVO4, 50 mM NaF, 1 mM PMSF, and 1% protease inhibitor cocktail (Sigma‐Aldrich). The protein concentration was determined using the Bradford method (Bio‐Rad). Cell fractions (25–30 μg/ml) were separated by SDS‐PAGE, transferred to PVDF membranes (Thermo Fisher), and incubated with primary antibodies (Table 1) overnight at 4⁰C followed by 1‐hour incubation with HRP‐conjugated secondary antibodies; Peroxidase‐AffiniPure Goat Anti‐Mouse IgG (Jackson ImmunoResearch/115–035–003) & Peroxidase AffiniPure Goat Anti‐Rabbit IgG (Jackson ImmunoResearch/111–035–003). Immunoreactive bands were visualized using a Bio‐Rad Imaging System (Image Lab software). Antibodies for α‐tubulin and TOMM20 were used as makers for cytosolic and mitochondria fractions, respectively.

**TABLE 1:** Information on antibodies used for biochemical studies

Antibody	Manufacturer/Catalog number	WB dilution	IF dilution	References
Primary antibodies				
ACBD3	Santa Cruz Biotech sc−101277	1:500	1:100	Teoule et al., 2013; Greninger et al., 2012; Smola et al., 2020)
D‐AKAP1	MyBioSource MBS8243156	1:800	1:100	Fernández‐Araujo et al., 2014)
D‐AKAP2	Abnova H00011216‐M04		1:100	Eggers et al., 2009)
D‐AKAP2	Santa Cruz Biotech sc−98755	1:500		
OPA1	Santa Cruz Biotech sc−393296	1:500	1:100	Huang et al., 2018; Kim et al., 2021; Koo & Kang, 2019; Xu et al., 2019; Li et al., 2019)
Rab32	Santa Cruz Biotech sc−390178	1:500		Kalogeropulou et al., 2020; Hu et al., 2019; Balci et al., 2020)
WAVE−1	Santa Cruz Biotech sc−271507	1:500		Moore et al., 2021; Vassilev et al., 2017)
SPHKAP	Invitrogen PA5‐27581	1:800		
PKA RI‐α/β	Santa Cruz Biotech sc−271125	1:500	1:50	Chen et al., 2017; Chiba et al., 2011)
PKA RII‐α	Santa Cruz Biotech sc−137220	1:500	1:50	Muñoz‐Llancao et al., 2017; Clister et al., 2019)
PKA RII‐β	Santa Cruz Biotech sc−376778	1:500	1:50	Just‐Borràs et al., 2020; Garcia et al., 2019; Chen et al., 2019)
PKA Cat‐α/β/γ	Santa Cruz Biotech sc−365615	1:500		Tibenska et al., 2020; Pérez‐Gómez & Tasker, 2013; Seward et al., 2013)
TOMM20	Cell Signaling Technology 42406	1:1000	1:100	Nie et al., 2020; Liu et al., 2019)
TOMM20	Abcam ab56783		1:100	Casey et al., 2000; Tavalin et al., 2002)
SERCA2	Invitrogen MA3‐919	1:1000		Rudokas et al., 2021; Hadipour‐Lakmehsari et al., 2019)
Na,K‐ATPase α1	Cell Signaling Technology 3010S	1:1000		Lou et al., 2017; Lichý et al., 2019)
Tubulin	Sigma Aldrich T5168	1:15000		
Secondary antibodies				
Goat anti‐mouse Alexa Fluor 568	Invitrogen A11004		1:1000	
Goat anti‐rabbit Alexa Fluor 647	Invitrogen A32733		1:1000	
Peroxidase‐AffiniPure Goat Anti‐Mouse IgG	Jackson ImmunoResearch 115–035–003	1:5000		
Peroxidase AffiniPure Goat Anti‐Rabbit IgG	Jackson ImmunoResearch 111–035–003	1:5000		

### 2.4 Immunocytochemistry and confocal imaging

Cardiac myocytes were plated on poly‐l‐lysine coated glass‐bottom plates for 1 h. These cells were then washed with PBS, fixed using 4% PFA for 15 min, and permeabilized with 0.2% Triton X‐100 for 20 min at room temperature. Cells were blocked in 1% BSA for 30 min followed by incubation with designated primary antibodies (Table 1) overnight at 4°C. The following day, the cells were washed with PBS and incubated with secondary antibodies Goat anti‐mouse Alexa Fluor 568 (Invitrogen/A11004) & Goat anti‐rabbit Alexa Fluor 647 (Invitrogen/A32733) for 1 h, followed by counterstaining with DAPI. The slides were refrigerated until imaged. Samples not treated with primary antibody were used to test for nonspecific binding of secondary antibodies (Figure S4).

Confocal images were obtained using an Olympus Fluoview 1000 microscope with an oil immersion objective (60X, 1.42 NA) and the following filter sets: DAPI (Ext: 405 nm, Ems: 430–470 nm), Alexa Fluor 568 (543 nm, 560–620 nm), and Alexa Fluor 647 (640 nm, 655–755 nm). The degree of fluorophore co‐localization was quantified by calculating the Pearson's correlation coefficient (PCC), which ranges from 1 for perfect co‐localization to −1 for perfect inverse co‐localization. The degree of fractional overlap of fluorophores was quantified by calculating the Mander's overlap coefficients (MOC) which range from 0 for no co‐localization to 1 for perfect co‐localization (Agarwal et al., 2016; Dunn et al., 2011). Autofluorescence due to excitation parameters at the wavelengths used in this study was negligible, and experiments were repeated in multiple cells from at least three different animals to confirm the consistency of fluorescence patterns.

## 3 RESULTS

### 3.1 Expression of mitochondrial AKAP mRNAs

**FIGURE 1:**
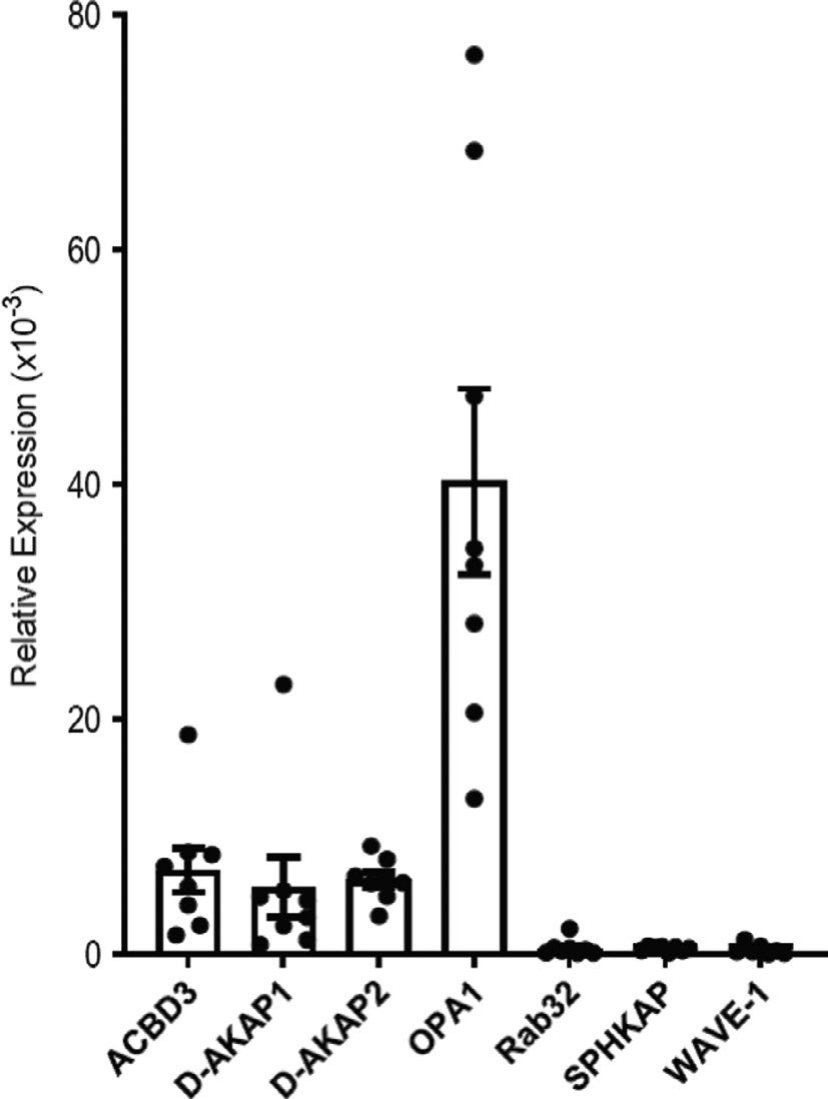
mRNA expression of mitochondria‐associated AKAPs in ARVM. Bar graphs show the mean ± S.E.M. expression value of AKAPs from ARVM normalized to the expression of the endogenous housekeeping gene, GAPDH; *n* = 7 independent RNA extractions analyzed in triplicate for each extraction

Previous studies have identified at least seven different AKAPs that are associated with mitochondria in various cell types: dual specific AKAP1 and 2 (D‐AKAP1 and D‐AKAP2), acyl‐CoA‐binding domain‐containing 3 (ACBD3), optic atrophy 1 (OPA1), WAVE‐1, Rab32, and sphingosine kinase type 1‐interacting protein (SPHKAP). To evaluate the relative expression level of mRNA for each of these AKAPs in ARVMs, we used the quantitative real‐time polymerase chain reaction (qPCR). The results indicate that the mRNA level of OPA1 was most abundant, followed by D‐AKAP1, D‐AKAP2, and ACBD3. The mRNA levels of WAVE‐1, Rab32, and SPHKAP were barely detectable (Figure 1).

### 3.2 Expression of mitochondrial AKAPs

**FIGURE 2:**
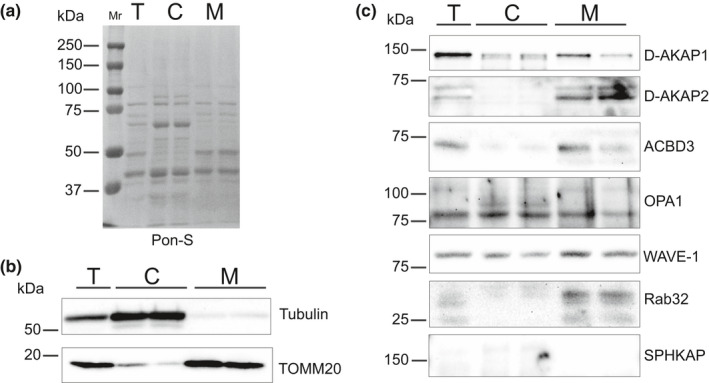
Expression levels of mitochondria‐associated AKAPs in ARVM. (a) Ponceau S (Pon‐S) staining as a loading control. Lanes represent total cell lysate (T), the cytosolic fraction (C), and the mitochondria enriched fraction (M) from ARVMs. (b) Tubulin was used as a marker for the cytosolic fraction and TOMM20 as the specific mitochondrial marker. (c) PVDF membrane was probed for indicated AKAPs. Molecular weight markers are placed adjacent to the blots. *n* ≥ 3 independent extractions

We then looked for the expression of mitochondrial AKAPs in total cell lysates, as well as cytosolic and mitochondrial cell fractions, using Western blot analysis (Figure 2a). The specificity of the antibodies used to probe for D‐AKAP1, D‐AKAP2, ACBD3, OPA1, WAVE‐1, and RAB32 was previously validated by knockdown experiments in other cell lines (Eggers et al., 2009; Fernández‐Araujo et al., 2014; Hu et al., 2019; Huang et al., 2018; Kalogeropulou et al., 2020; Kim et al., 2021; Moore et al., 2021; Teoule et al., 2013). Tubulin and TOMM20 were used as markers for the cytosolic and mitochondrial cell fractions, respectively (Figure 2b). To verify proper mitochondrial enrichment and assess collected fraction for contamination, we further probed our samples for Na,K‐ATPase α1 and the sarco‐endoplasmic reticulum Ca^2+^ ATPase 2 (SERCA2) as markers for surface membrane and the sarcoplasmic reticulum, respectively (Figure S1). Consistent with our qPCR data, we found evidence for the protein expression of all AKAPs except for SPHKAP in the total cell lysates (Figure 2c). We also detected evidence for the protein expression of Rab32 and WAVE‐1, despite finding little mRNA (Figure 1). Upon further analysis, D‐AKAP1, OPA1, and WAVE‐1 were found in both cytosolic and mitochondrial fractions, while D‐AKAP2, ACBD3, and Rab32 were primarily found in mitochondria enriched fractions. D‐AKAP1, ACBD3, and Rab32 appeared as single bands at the expected molecular weight (Das Banerjee et al., 2017; Gabrovsek et al., 2020; Hu et al., 2019; Kacal et al., 2021; Kalogeropulou et al., 2020; Tan et al., 2021; Teoule et al., 2013). The identification of multiple immunoreactive bands for D‐AKAP2 may be explained by the existence of splice variants as observed in mouse brain and lung extracts (Wang et al., 2001). The same is true for OPA1, which also shows multiple splice variants (Del Dotto et al., 2017; Haushalter et al., 2019).

### 3.3 Mitochondrial colocalization of AKAPs

**FIGURE 3:**
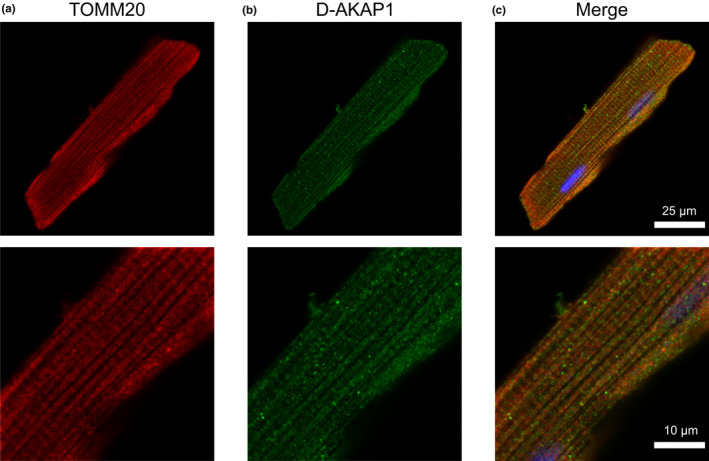
Co‐localization of D‐AKAP1 and mitochondria in ARVM. Representative confocal images of ARVM showing mitochondrial marker (a) TOMM20 and (b) D‐AKAP1. (c) Merged image of TOMM20 (red), D‐AKAP1 (green), and DAPI (blue) shows that D‐AKAP1 overlaps the mitochondrial marker. PCC = 0.77 ± 0.03; *N* = 4 independent isolations with *n* = 6 from each isolation. Magnified images are shown below each corresponding image with the appropriate scale bar

**FIGURE 4:**
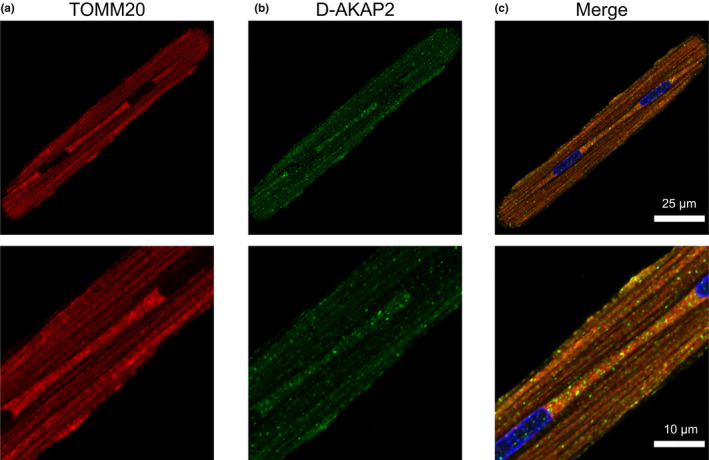
Co‐localization of D‐AKAP2 and mitochondria in ARVM. Confocal images of ARVM showing mitochondrial marker (a) TOMM20 and (b) D‐AKAP2, presenting a punctate pattern and overlap with mitochondria. (c) Merged images representing TOMM20 (red), D‐AKAP2 (green), and DAPI (blue). PCC = 0.72 ± 0.03; *N* = 4 independent isolations with *n* = 6 from each isolation. Magnified images are shown below each corresponding image with the appropriate scale bar

**FIGURE 5:**
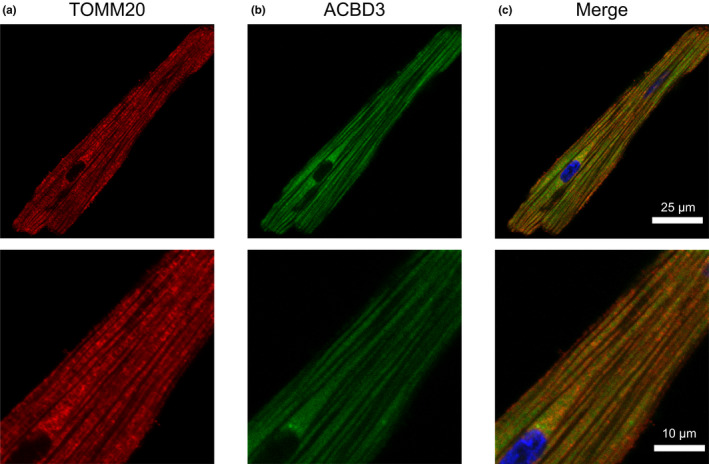
Co‐ labeling of ACBD3 with mitochondria in ARVM. Labeling of the mitochondrial marker, (a) TOMM20 and (b) ACBD3 show a similar pattern of immunoreactivity. (c) Merged images of TOMM20 (red), ACBD3 (green), and DAPI (blue) show that ACBD3 is distributed in a diffuse manner generating a PCC of 0.56 ± 0.02; *N* = 4 independent isolations with *n* = 6 from each isolation. Magnified images are shown below each corresponding image with the appropriate scale bar

**FIGURE 6:**
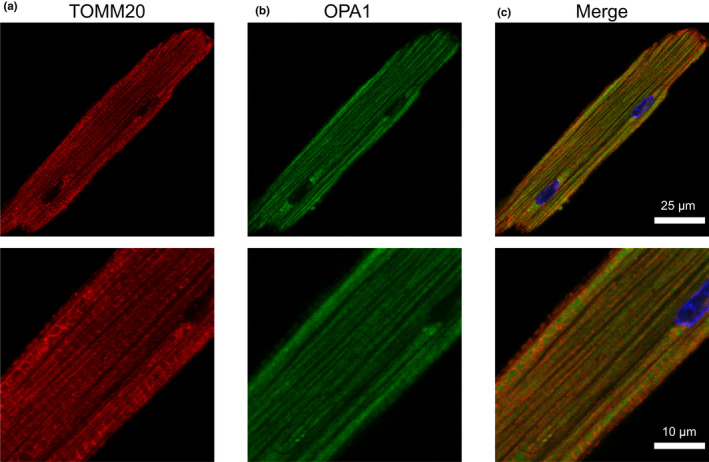
Co‐ labeling of OPA1 with mitochondria in ARVM. Labeling of the mitochondrial marker, (a) TOMM20 and (b) OPA1. (c) Merged images of TOMM20 (red), OPA1 (green), and DAPI (blue). PCC = 0.58 ± 0.03; *N* = 4 independent isolations with *n* = 6 from each isolation. Magnified images are shown below each corresponding image with the appropriate scale bar

Based on the results of our immunoblot analysis, we then used immunocytochemistry to verify whether these AKAPs co‐localize with mitochondria in ARVMs. It should be noted that the goal of these experiments was not to demonstrate a specific interaction of these AKAPs with TOMM20, but rather to assess the degree of association with the outer mitochondrial membrane, where TOMM20 is expressed. Co‐staining of each AKAP with TOMM20 demonstrated that D‐AKAP1, D‐AKAP2, ACBD3, and OPA1 localize to mitochondria, consistent with our immunoblot findings in subcellular fractions. In order to quantify the degree of colocalization between AKAPs and mitochondria, we calculated Pearson's correlation coefficient (PCC). The PCC values for colocalization with TOMM20 were: D‐AKAP1 (0.77 ± 0.02, Figure 3), D‐AKAP2 (0.72 ± 0.02, Figure 4), ACBD3 (0.56 ± 0.02, Figure 5), and OPA1 (0.58 ± 0.03, Figure 6). Based on these values, we assume moderate to strong colocalization of the AKAPs with the mitochondria. Determination of the fractional overlap was mathematically expressed through the Mander's overlap coefficient (MOC), which compares the co‐occurrence of fluorescence among pixels. In contrast to PCC, MOC represents the extent to which two probes occupy the same spatial region (Adler & Parmryd, 2010; Dunn et al., 2011). Our primary interest was in the fraction of AKAP found in regions positive for mitochondria, for which the MOC values were: D‐AKAP1 (0.87 ± 0.03, Figure 3), D‐AKAP2 (0.83 ± 0.03, Figure 4), ACBD3 (0.76 ± 0.03, Figure 5), and OPA1 (0.78 ± 0.03, Figure 6). Overall, our results suggest that D‐AKAP1, D‐AKAP2, ACBD3, and OPA1 co‐localize with the mitochondria to a high degree. However, the small fraction of each that does not overlap with mitochondria and may represent the fact that these AKAPs have also been associated with other cellular structures (see Discussion).

Attempts to obtain immunofluorescence signals using Rab32 and WAVE‐1 antibodies were not successful (Figures S2 and S3). The Rab32 antibody has been used with immunofluorescence techniques in cell lines (Balci et al., 2020; Hu et al., 2019), so levels of Rab32 might not have been enough for detection in isolated ARVMs. The WAVE‐1 antibody is specific for epitopes near the N‐terminal domain, which in part, regulates subcellular localization of the protein and could potentially be masked to the antibody. Ectopic expression of tagged Rab32 and WAVE‐1 would be a potential avenue to get a better idea of their subcellular localization with an immunofluorescence‐based technique in a single cell resolution.

### 3.4 Protein expression level of PKA in mitochondria

**FIGURE 7:**
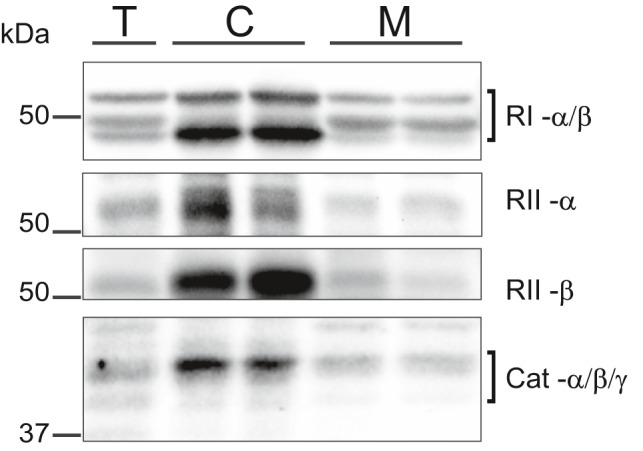
Expression levels of PKA subunits in mitochondria. Exemplar immunoblot for the expression of PKA regulatory, RIα/β, RIIα, RIIβ, and catalytic (Cat‐α/β/γ) subunits are shown in total cell lysate (T), the cytosolic fraction (C), and mitochondria enriched fraction (M); n ≥ 3 independent protein extractions

Most AKAPs bind specifically to type II PKA regulatory (RII) subunits. However, ACBD3 binds specifically to RI subunits, while D‐AKAP1 and D‐AKAP2 can bind both RI and RII subunits. To this end, we probed subcellular fractions with the antibodies directed against the PKA regulatory subunit isoforms RIα/β, RIIα, and RIIβ as well as the associated catalytic (Cat‐α/β/γ) subunit (Figure 7). The antibody we used to detect RI subunits did not distinguish between the α or β isoforms, while RIIα and RIIβ were probed using separate antibodies. Western blot analysis of total cell lysates showed evidence of both RI subunits with the α isoform observed at ~47 kDa while the β isoform was at ~51 kDa, both in the cytosolic as well as the mitochondrial fraction. RIIα and RIIβ also presented similar immunodetection patterns as RI subunits in the total, cytosolic and mitochondrial fraction of ARVMs. Overall, our Western blot data confirmed that all PKA subunits, RIα/β, RIIα, RIIβ, and Cat‐α/β/γ can be found in the mitochondrial fraction of ARVMs.

### 3.5 Mitochondrial colocalization of PKA regulatory subunits

**FIGURE 8:**
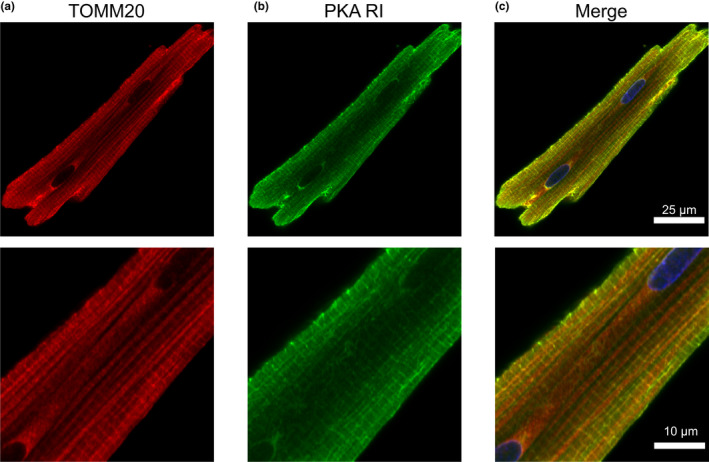
PKA RI subunit and mitochondria co‐labeling. Representative confocal images of ARVM showing mitochondrial marker (a) TOMM20 and (b) PKA RI regulatory subunit. (c) Composite image showing strong overlap of TOMM20 (red) and PKA RI subunit (green) in the subsarcolemmal mitochondria. PKA RI antibody is also seen to label transverse striations. PCC = 0.69 ± 0.02, MOC = 0.80 ± 0.03; *N* = 4 independent isolations with *n* = 6 from each isolation. Magnified images are shown below each corresponding image with the appropriate scale bar

**FIGURE 9:**
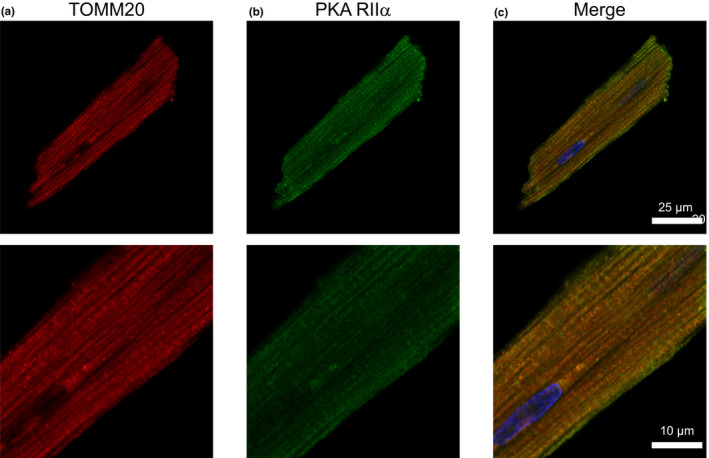
Co‐localization of PKA RIIα subunit with mitochondria. Representative confocal images of ARVM showing mitochondrial marker (a) TOMM20 and (b) PKA RII‐α subunit. (c) Merged images of TOMM20 (red), PKA RIIα subunit (green), and DAPI (blue). PCC = 0.63 ± 0.02, MOC = 0.80 ± 0.02; *N* = 4 independent isolations with *n* = 6 from each isolation. Magnified images are shown below each corresponding image with the appropriate scale bar

**FIGURE 10:**
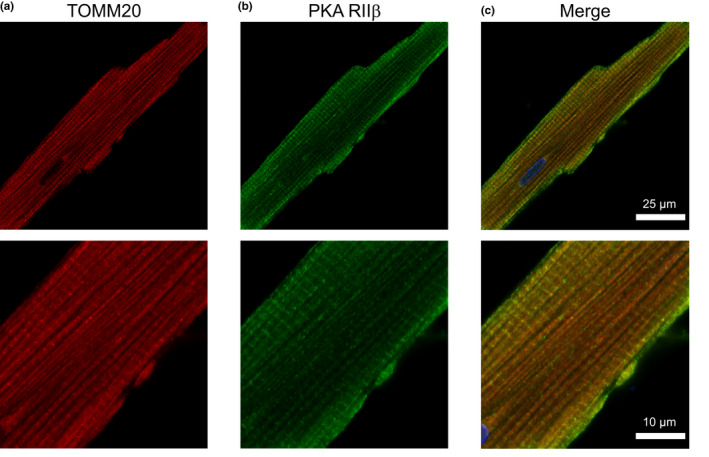
Co‐localization of PKA RIIβ subunit with mitochondria. Representative confocal images of ARVM showing mitochondrial marker (a) TOMM20 and (b) PKA RII‐β subunit. (c) Merged image shows moderate colocalization between TOMM20 (red), PKA RII‐β (green), and DAPI (blue). PCC = 0.61 ± 0.02, MOC = 0.73 ± 0.02; *N* = 4 independent isolations/experiments with *n* = 6 from each isolation. Magnified images are shown below each corresponding image with the appropriate scale bar

To corroborate our Western blot data on the distribution of different PKA subunits, we performed confocal imaging of ARVMs co‐labeled with antibodies against the PKA R subunits and TOMM20. The PCC values represented strong colocalization between the R subunits and mitochondria: RIα/β (0.69 ± 0.02, Figure 8), RIIα (0.63 ± 0.02, Figure 9), and RIIβ (0.61 ± 0.02, Figure 10). MOC values for the fraction of PKA R subunits found in regions positive for mitochondria was ≥0.73 for each R subunit tested. The results demonstrated that a considerable fraction of the three regulatory subunit antibodies co‐localized with mitochondria marker TOMM20.

In adult cardiac myocytes, the mitochondrial population can be defined according to their location, identifiable by electron microscopy. The subsarcolemmal mitochondria lie just beneath the sarcolemma and are thought to be involved in ion homeostasis or signaling pathways. In comparison, the intermyofibrillar mitochondria are highly ordered between rows of contractile proteins and thought to supply energy for myosin and sarcoplasmic reticulum localized ATPases (Piquereau et al., 2013; Porter et al., 2011). Higher magnification images show that among the regulatory subunits, PKA RI‐α/β displayed a generally diffuse signal with higher intensity at the edges of the cell where one would expect to find subsarcolemmal mitochondria. This is similar to mouse ventricular myocytes where PKA RIα was observed to be enriched within the subsarcolemmal mitochondrial fractions of heart tissue (Haushalter et al., 2019). Also, PKA RI‐α/β displayed a prominent transverse striated labeling pattern. For PKA RIIα, we observed a staining pattern more consistent with TOMM20, while PKA RIIβ labeling displayed a mix of mitochondrial colocalization and transverse striations as seen with PKA RI‐α/β. Studies have shown other AKAPs, such as AKAP18δ exhibit a striated pattern overlapping α‐actinin labeling and form a SERCA2–PLN–AKAP18δ–PKA complex in rat cardiac myocytes (Henn et al., 2004; Lygren et al., 2007). mAKAP and Synemin are cardiac AKAPs that localize to the Z‐line and anchor PKA RI and RII, respectively (Greninger et al., 2012; Schaper et al., 1985).

## 4 DISCUSSION

### 4.1 Expression of mitochondrial AKAPs in ARVMs

In the present study, our qPCR data suggest that D‐AKAP1, D‐AKAP2, ACBD3, and OPA1 are the predominant mitochondrial AKAP isoforms expressed in ARVMs. Furthermore, Western blot analysis indicated that there is a significant expression of these AKAPs at the protein level in mitochondrial fractions of the cell, and immunocytochemistry confirmed that they exhibit strong co‐localization with mitochondria. Despite relatively low message levels, we also detected some evidence for the expression of Rab32 and WAVE‐1 protein by Western blot. However, we failed to see evidence for significant expression in immunolabeled images (Figures S2 and S3). Also, we found little evidence for the expression of SPHKAP at either the message or protein level. These results suggest that D‐AKAP1, D‐AKAP2, ACBD3, and OPA1 are the main AKAP isoforms associated with mitochondria in ARVMs.

D‐AKAP1 is a dual‐specific AKAP that contains a targeting motif that anchors it to the cytoplasmic face of the outer mitochondrial membrane. However, alternative splicing has also been shown to result in the targeting of this protein to the endoplasmic reticulum (Huang et al., 1997, 1999). In the present study, we detected a single D‐AKAP1 immunoreactive band at ~121 kDa in both cytosolic and mitochondrial fractions of the cell (Figure 2c). Furthermore, our immunolabeling experiments indicate that D‐AKAP1 exhibits a high degree of colocalization with mitochondria (Figure 3). D‐AKAP2 is another dual‐specific AKAP that anchors PKA to the outer mitochondrial membrane, at least in some cell types, including mouse cardiac myocytes (Wang et al., 2001). We confirmed this finding in ARVMs with our Western blot experiments, where we observed two prominent immunoreactive bands found exclusively in mitochondrial fractions of the cell (Figure 2c). The presence of multiple bands may represent splice variants, which have been reported in other tissues (Eggers et al., 2009; Wang et al., 2001). Specific targeting to mitochondria was confirmed by the colocalization of D‐AKAP2 with TOMM20 in imaging experiments (Figure 4).

D‐AKAP1 & 2 have been subjects of interest due to their role in cardiac morphology and function. Studies of D‐AKAP1 genetic deletion indicate that it plays a role in limiting the development of cardiac hypertrophy (Schiattarella et al., 2018). In neonatal cardiac myocytes, knockdown of D‐AKAP1 induced significant hypertrophy and in adult mice, deletion of the D‐AKAP1 exhibited exacerbation of cardiac hypertrophy in response to pressure overload (Schiattarella et al., 2018). For D‐AKAP2, genetic polymorphism has been associated with an increased incidence of heart disease, and in mice, this mutation was found to produce conduction abnormalities associated with changes in the PR interval of the electrocardiogram (Kammerer et al., 2003). Overall the function of AKAPs extends beyond just anchoring PKA, but it is plausible to speculate that the dysfunctional outcomes due to AKAP malfunction are manifestations of disrupting cAMP buffering.

In the case of ACBD3, a type I PKA (RI) specific AKAP, it has been linked with several cellular activities, including steroid biosynthesis (Li et al., 2001). In addition to being found in the outer membrane of mitochondria, ACBD3 has also been associated with the Golgi (Li et al., 2001; Sohda et al., 2001). Previous studies have demonstrated that ACDB3 can be found in cardiac tissue (Li et al., 2001; Liu et al., 2003). In the present study, we demonstrate that ACBD3 is expressed in isolated cardiac myocytes, where it is concentrated in the mitochondrial fraction (Figure 2c) and co‐localizes with mitochondria (Figure 5).

OPA1 is a dynamin‐like protein that has been studied extensively because of its role in regulating mitochondrial dynamics (Del Dotto et al., 2017). Previous studies have suggested the possibility that OPA1 undergoes post‐transcriptional modification (Herlan et al., 2003; McQuibban et al., 2003; Sesaki et al., 2003). Our Western data support this idea with the detection of both long and short isoforms in mitochondrial fractions (Figure 2c). OPA1 has also been reported to function as a dual‐specific AKAP associated with lipid droplets (Pidoux et al., 2011). This may explain the presence of OPA1 immunoreactive bands in cytosolic fractions. However, in mitochondria, OPA1 is restricted to the inner mitochondrial membrane (Olichon et al., 2002), which suggests that it is unlikely to play a role in anchoring PKA regulatory subunits involved in limiting cytosolic diffusion of cAMP (Tibenska et al., 2020).

As mentioned before, we were not able to obtain immunofluorescence signals from Rab32 and WAVE‐1 antibodies (Figures S2 and S3). Previous success with the use of Rab32 antibody in cell lines suggests that there may be very low levels of Rab32, unsuitable for detection in single ARVMs. Issues with immunogenic epitope recognition could have prevented immunofluorescence‐based detection as the antibody specific for WAVE‐1 targets the epitopes near the N‐terminal domain. This region regulates the subcellular localization of the protein and also binds with phosphatidylinositol (4, 5)‐bisphosphate and other anionic phospholipids (Miki et al., 1998; Pollitt & Insall, 2009; Suetsugu et al., 1999). Due to these reasons, the epitopes might not be accessible to the antibodies in formalin‐fixed cells but are more readily detectable under denatured conditions during the Western blotting procedure.

### 4.2 Association of PKA regulatory subunits with mitochondria in ARVMs

A quintessential role for AKAPs is to anchor the PKA holoenzyme in close proximity to its effectors to ensure appropriate phosphorylation. However, AKAP binding of PKA regulatory subunits may play other important roles as well, especially when one considers the fact that the number of regulatory subunits far exceeds the number of catalytic subunits. Walker‐Gray et al. (Walker‐Gray et al., 2017) reported that the number of regulatory subunits is more than sixfold greater than the number of catalytic subunits in the rat heart. This observation is significant for multiple reasons. In addition to ensuring rapid catalytic subunit re‐association, an excess of regulatory subunits, especially those bound to AKAPs, may contribute significantly to the buffering capacity for cAMP, slowing its diffusion throughout the cell.

We have previously demonstrated that the diffusion coefficient of cAMP in ARVMs is significantly slower than that expected assuming free diffusion (Agarwal et al., 2016). Loading these cells with fluorescently labeled cAMP produces a distinct pattern due to co‐localization with mitochondria. Furthermore, blocking AKAP‐PKA regulatory subunit interactions disrupts this pattern and increases the rate of cAMP diffusion. These results suggest that the slow diffusion of cAMP is due at least in part to the buffering effect of PKA bound to the outer mitochondrial membrane (Agarwal et al., 2016). Our present results suggest that D‐AKAP1, D‐AKAP2, and ACBD3 are most likely the principal AKAPs responsible for anchoring PKA regulatory subunits to the outer surface of mitochondria in ARVMs. Future studies should focus on knocking down the said mitochondrial AKAPs, individually or as a group, and assessing functional effects as well as potential changes in the diffusion coefficients of PKA regulatory subunits or cAMP.

Another critical question is then, what types of regulatory subunits are associated with those AKAPs? Our results indicate that both RI and RII subunits can be found in mitochondrial fractions and co‐localize with mitochondria. A previous study showed that PKA RI subunits outnumber RII subunits 2 to 1 in rat heart (Walker‐Gray et al., 2017). Our initial investigation of the contribution of regulatory subunits in buffering cAMP movement only considered RII subunits (Agarwal et al., 2016). However, by disregarding the potential role of RI subunits anchored to the outer mitochondrial membrane by either of the dual specific AKAPs, D‐AKAP1 and D‐AKAP2, or the RI specific ACBD3, we may have significantly underestimated the effect of PKA‐dependent buffering of cAMP mobility. Future studies should take into consideration the fact that RI subunits are likely to play at least as important a role as RII subunits.

Another question yet to be answered is, what proportion of RI and RII subunits are not associated with catalytic subunits? This becomes important when one considers the fact that the cAMP affinity of free RI subunits is approximately 1.3 nM, while that of the holoenzyme is 2.9 μM (Dao et al., 2006). With a basal cAMP concentration in the vicinity of 1 μM (Iancu et al., 2008), one would predict that free regulatory subunits may play a particularly important role in buffering the movement of cAMP throughout the cell. For the field of cAMP compartmentation, a rigorous investigation of PKA regulatory units and their state of assembly at different subcellular locations would be important, especially those anchored to the surface of mitochondria.

### 4.3 Conclusion

Compartmentation of cAMP signaling is critical for maintaining the integrity of receptor‐specific responses, and it has been hypothesized that slow diffusion of cAMP, independent of PDE activity, plays an important role in that process (Agarwal et al., 2016; Feinstein et al., 2012; Iancu et al., 2007; Lohse et al., 2017; Saucerman et al., 2006, 2014; Yang et al., 2016; Zhang et al., 2020). Furthermore, our previous work has demonstrated that buffering or binding of cAMP by PKA regulatory subunits anchored to mitochondria plays a major role in slowing cAMP diffusion in cardiac myocytes (Agarwal et al., 2016).

Mitochondria take up approximately 30% of the cell volume in an adult ventricular myocyte, where they are packed tightly beneath the plasma membrane as well as around the transverse tubule network and contractile proteins (Barth et al., 1992; Schaper et al., 1985). Because mitochondria are impermeable to cAMP, they form a network of physical barriers that cAMP must negotiate. Add to that the fact that PKA RI and RII subunits, either alone or as part of a holoenzyme complex, are anchored to the mitochondrial surface by D‐AKAP1, D‐AKAP2, and ACBD3, suggests that this arrangement creates an ideal system for limiting the movement of cAMP throughout the cell.

## CONFLICT OF INTEREST

No conflicts of interest are declared by the authors.

## AUTHOR CONTRIBUTIONS

RTS, CF, KM, SRA, and RDH conceived and designed the research; RTS, CF, KM, MWR, and AW performed experiments; RTS and KM prepared figures; RTS drafted the manuscript and KM, SRA, and RDH edited and revised the manuscript. RTS, CF, KM, MWR, SRA, and RDH approved the final version of the manuscript.

## Supporting information



Supplementary Material
