# Supplementary material for: Mitochondrial A‐kinase anchoring proteins in cardiac ventricular myocytes
Source: Physiol Rep. 2021 Sep 13;9(17):e15015. doi: 10.14814/phy2.15015 (PMC8436057; doi:10.14814/phy2.15015)
Supplement: Supplementary file 1 — Supplementary Material [file PHY2-9-e15015-s001.pdf]

Supplemental Table S1. Primer sequences for AKAPs and GAPDH:

**Gene**	**GeneID**	**Forward (5'-3')**	**Reverse (5'-3')**
ACBD3	NM_182843.1	AGTGAGTCCAGTGACGACGA	TCATCCAGCAGAGGCTTGTT
D-AKAP1	NM_053665.1	ACTGAGCCCTTCAGCAATGG	AGCCCACAACAGCTATCCAC
D-AKAP2	NM_001114606.1	TGGAACTTCGGAGAATGGAGC	TGTGTGCTCTTATTCGGGACC
OPA1	NM_133585.3	CCGCTTCATGACAGAACCCA	TGTGCCGCTTGATACTCTCC
Rab32	NM_001108902.1	ACTTCGCCCTCAAAGTCCTC	GTCATGTTGCCAAACCGCTC
SPHKAP	NM_001127492.1	CCAATGAGGAGGACAACCCAG	TAATGCCAGACTGCTAGTGCC
WAVE-1	NM_001025114.1	GTACCCGTTCATCCACTCCC	CGACCGTGACAGGTTATGAT
GAPDH	NM_017008.4	GACATGCCGCCTGGAGAAAC	AGCCCAGGATGCCCTTTAGT

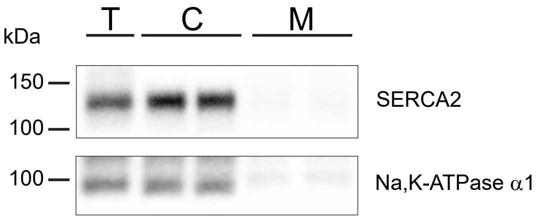

Supplementary Figure S1. Verification of mitochondrial enrichment.
Lanes representing total cell lysate (T), the cytosolic fraction (C), and the mitochondria enriched fraction (M) from ARVMs, were probed with as Na,K-ATPase α 1 as a marker for the surface membrane and the Sarco-/ER Ca^{2+} ATPase 2 (SERCA2) for the sarcoplasmic reticulum, as confirmation of the purity of the mitochondrial fraction. Molecular weight markers are placed adjacent to the blots. n = 4 independent extractions.

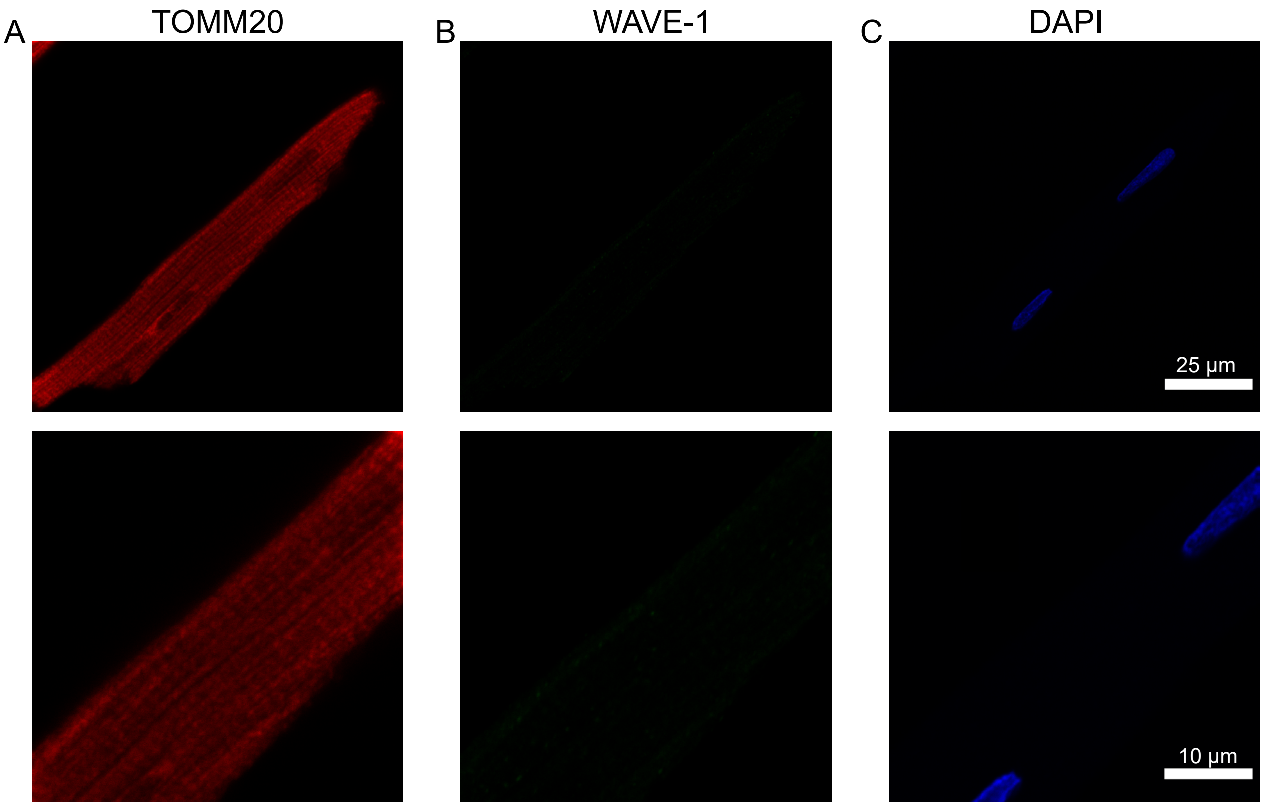

Supplementary Figure S2. Co- labeling of WAVE-1 with mitochondria in ARVM. Labeling of the mitochondrial marker, A) TOMM20, B) WAVE-1, and C) DAPI. Magnified images are shown below each corresponding image with the appropriate scale bar.

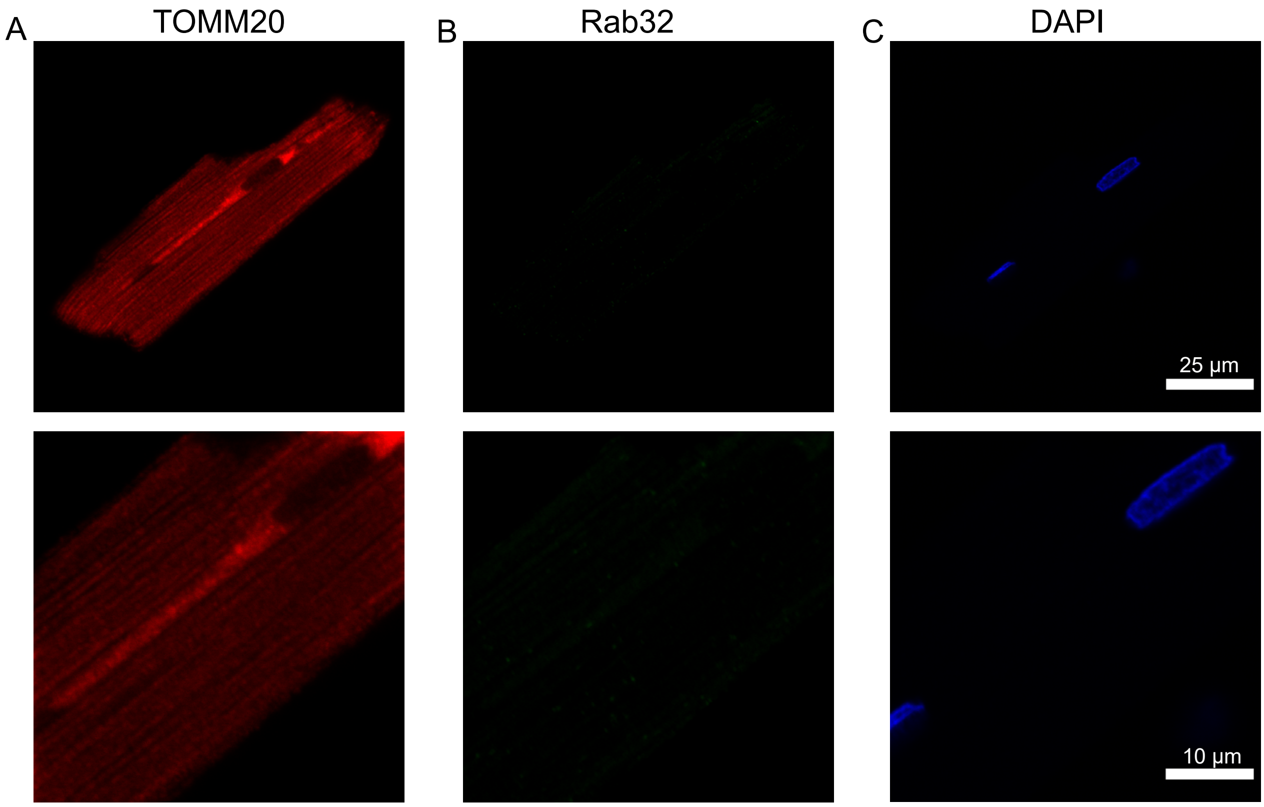

Supplementary Figure S3. Co- labeling of Rab32 with mitochondria in ARVM. Labeling of the mitochondrial marker, A) TOMM20, B) Rab32, and C) DAPI. Magnified images are shown below each corresponding image with the appropriate scale bar.

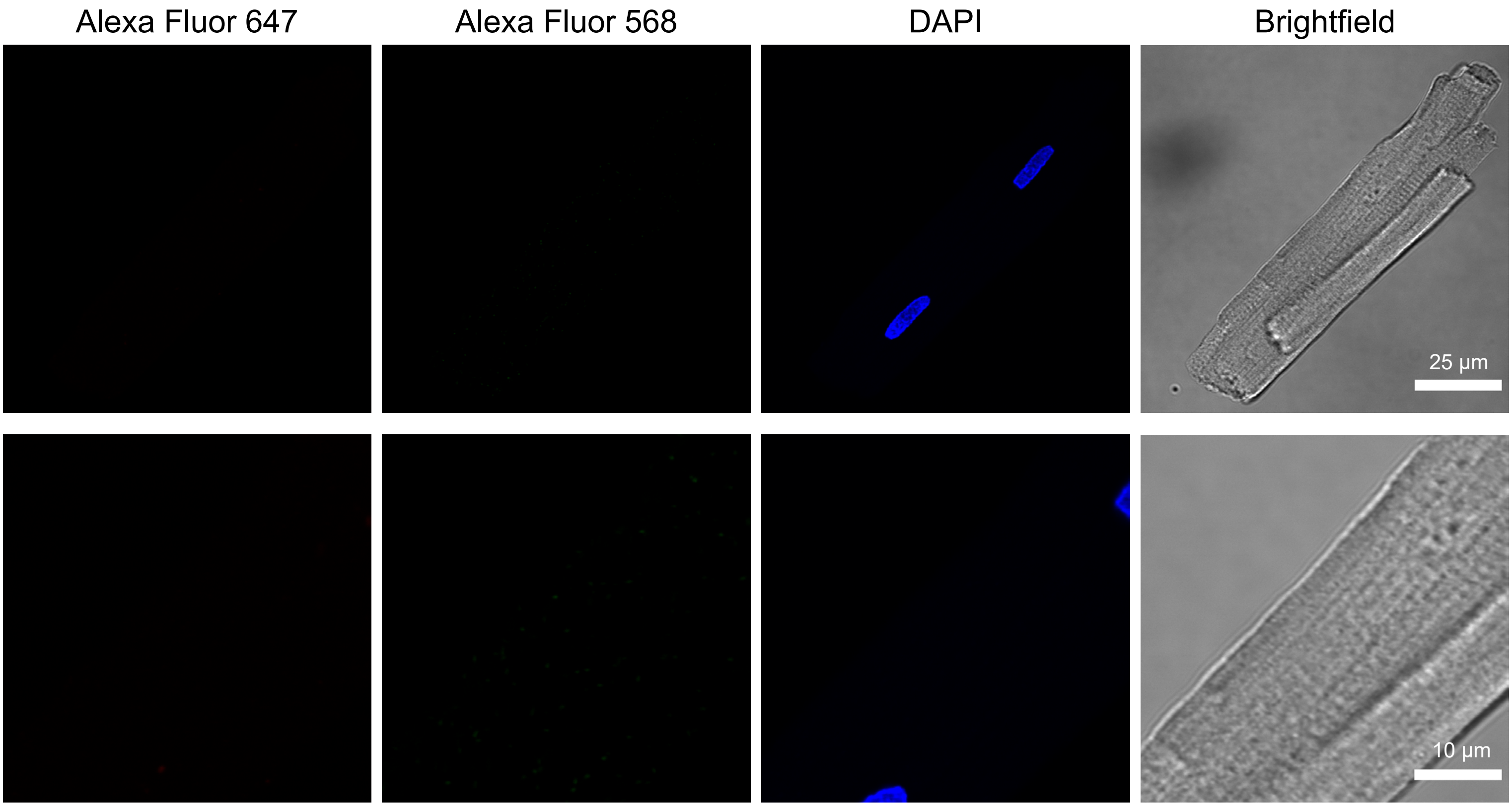

Supplementary Figure S4. No primary control immunofluorescence images from ARVM.
ARVMs processed under the same conditions for immunofluorescence but without primary antibody addition exhibit negligible signal. Panels show confocal scans of Alexa 647, Alexa 568, DAPI, and brightfield. Magnified images are shown below each corresponding image with the appropriate scale bar.
